# Trending Anti-E7 Serology Predicts Mortality and Recurrence of HPV-Associated Cancers of the Oropharynx

**DOI:** 10.1155/2022/3107990

**Published:** 2022-09-26

**Authors:** Luke Johnson, Dat T. Ha, Melissa B. Hall, Gregory Shoemaker, Paul A. Bevins, John Strickley, Shadmehr Demehri, Rebecca A. Redman, Joongho Joh

**Affiliations:** ^1^Department of Medicine, University of Louisville School of Medicine, KY 40202, USA; ^2^Brown Cancer Center, University of Louisville School of Medicine, KY 40202, USA; ^3^John T Milliken Department of Internal Medicine, Washington University School of Medicine in St. Louis, MO 63110, USA; ^4^Center for Cancer Immunology, Center for Cancer Research, Massachusetts General Hospital and Harvard Medical School, Boston, MA 02114, USA; ^5^Cutaneous Biology Research Center, Department of Dermatology, Massachusetts General Hospital and Harvard Medical School, Boston, MA 02114, USA

## Abstract

High-risk human papillomavirus (HPV) is among the most common causes of head and neck cancer (HNC) with increasing incidence. HPV-associated HNC patients' clinical response to treatment varies drastically, which has made treatment de-escalation clinical trials challenging. To address the need for noninvasive biomarkers that differentiate patient outcomes, serum antibodies to E7 oncoprotein levels were evaluated in serial serum specimens from HPV-positive HNC patients (*n* = 48). We have found that increasing antibodies to E7 throughout treatment correlates with increased cancer recurrence or progression to mortality (*p* = .004) with 100% specificity as a predictive test.

## 1. Introduction

Head and neck cancer (HNC) is estimated to affect over 4 million people worldwide and is the seventh most common cancer type [1–3]. Human papillomavirus (HPV)-associated HNC has been on the rise for the last 3 decades and surpassed cervical cancer as the most prolific cause of HPV-associated cancer in the United States [1–3]. Although it is diagnosed at a median age of 60 years, a recent rise in HPV-associated HNC has increased the HNC incidence in younger patients [2, 3].

The most common type of HNC is squamous cell carcinoma (HNSCC). The risk factors for HNSCC include smoking and high-risk HPV [4]. Although smoking prevalence has been on the decline for the past half century, HPV-associated HNC remains a significant threat [4]. 50% of HNC patients have detectable oncogenic HPV DNA, with the highest proportion of HPV positivity in non-smokers [4]. HPV positivity may guide the treatment strategies as T-cell-specific therapies have been reported to be more effective in HPV-positive patients [5–7]. Current clinical guidelines for HNC patients outline an initial p16 test with a follow-up HPV DNA test [8]. However, p16 immunohistochemistry has been widely accepted as a surrogate marker for HPV essentially replacing HPV in situ hybridization (ISH) in clinical settings [9]. The reason for this is that HPV ISH is not done routinely in practice and sometimes includes low-risk HPV subtypes. Therefore, because of the availability of p16 data for each patient, this marker was used to identify HPV-associated cancers in this manuscript.

Although the benefit of identifying p16 status is evident, there is significant heterogeneity within HPV-associated HNC and patients' treatment response varies drastically [10]. There are currently limited molecular tools for differentiating between treatment responders and treatment non-responders. Multiple recent clinical trial investigation de-escalation treatment strategies have failed to meaningfully change clinical practice [10].

Therefore, this study sought to identify molecular markers for squamous cell head and neck cancer (HNSCC) that can be used to tailor cancer treatment to individual patient needs. Our research has been particularly interested in two potential prognostic markers: oncoproteins E6 and E7, which inactivate the tumor suppressors p53 and RB, respectively [11]. P16, the HPV-associated HNC marker used in this paper, is upregulated by E7's suppression of RB [12, 13].

This presence of E7 in the blood stream when cancer is active is due to one of the following mechanisms: cancer cells floating in the blood are transcriptionally active, tumors, release oncoproteins directly into tumor vascular beds because of necrosis, or tumor cells are secreting exosomes of viral oncoproteins [14–17]. Therefore, measuring antibodies to E7 via liquid biopsy throughout the treatment course provides a potential marker for predicting cancer outcome and activity. Here, we aim to further examine the activity of E7 throughout cancer, the potential of E7 antibodies to be used to monitor cancer, and its potential as a noninvasive prognostic marker.

## 2. Methods

The study was reviewed and approved by the Institutional Review Board at the University of Louisville (IRB# 08.0388, 15.0582) in compliance with the Declaration of Helsinki. De-identified patient sera and clinical information were obtained from the Clinical Trials Office Biorepository of the Brown Cancer Center. Blood was drawn for serum analysis prior to treatment and every 3 months thereafter for 2 years. All serum specimens were obtained by collecting blood into nonadditive vacutainers, processed by centrifugation after a 30-minute clot, aliquoted, and stored at 4°C until analyzed.

Human E7 oncoprotein was created from a viral plasmid (pQE30) in bacterial cell culture from the manufacturer (Qiagen). The production of E7 oncoprotein was confirmed via gel electrophoresis and Western Blot. This same process of E7 antigen production is described in more detail in our previous manuscript [18]. The ELISA 96 plates (Immulon 2HB) were purchased from the manufacturer (Thermo Sci). The ELISA tray was coated with E7 oncoprotein at 1:200 ratio with PBS for 1 hour and washed with ×3. Patient serum at a 1:50 ratio with PBS was added to the ELISA tray overnight. In the morning, the tray was washed ×3. Secondary mouse anti-human antibodies with alkaline phosphatase conjugate (Sigma) were added at a 1:2000 ratio for 1 hour and then washed with ×3. The signal was developed in the ELISA tray by adding alkaline phosphatase per ELISA manufacturer instructions. The ELISA assays were then read via Synergy HT (BioTek) at time intervals 30, 50, and 70 minutes. 50 minutes was used as the reporting data as it provided the clearest signal differentiation between test and controls.

HPV-associated HNC patients were identified with p16 immunohistochemistry. All patients had cancers of the oropharynx with exception of patients 881 and 891. Patient 881 had a cancer of the larynx, and patient 891 had cancer of the oral tongue. Anti-E7 positive patients were identified with the standard ELISA cutoff procedure: at least one visit where the mean ELISA value was more than the mean of the negative control value +3 standard deviations (.545 for HPV-16 anti-E7 and .423 for HPV-18 anti-E7) which was established in our p16 negative patients (750, 865, 872, and 1002). If the patient did not meet this criterion, they were classified as negative. Increasing trends were identified as at least 1 increasing trend line throughout the study period for high-risk anti-E7. Decreasing trending patients were defined as having no increasing trend and at least 1 decreasing trend for high-risk anti-E7.

The data was analyzed utilizing the Microsoft Excel, Prism, and MedCalc statistical software. The analysis of significance was done via FISHER's exact testing of contingency tables as the cohort was too small for a chi-square analysis to be accurate. For contingency tables larger than 2 × 2, a Freeman-Halton extension was added to the FISHER's exact test.

## 3. Results

Of the 48 patients in this study, our ELISA results indicate that 45.8% are positive for high-risk anti-E7 at one or more collection time points during the study period with positivity being defined as +3 standard deviations above the mean of the p16 negative patients ELISA values. Of the 43 patients available for follow-up, 56% were aged less than 60, 86% were male, 95% were white, and 5% were African American. The majority (91%) of patients were stage 3 or 4 at diagnosis. The demographic and clinical characteristics of the patients can be seen in supplementary table 1. Treatments underwent by patients primarily were comprised of surgery followed by immunotherapy, chemotherapy, and radiation. Specific treatments chosen for each patient can be seen in supplementary table 2. Eight patients had relapse of cancer with metastasis to various sites including the lungs, pericardium, lymph nodes, brain, bone, and liver. Nine patients suffered mortality, and 8 patients suffered cancer recurrence. The patients suffering recurrence and mortality are shown and separated by HPV anti-E7 trend status in supplementary table 3. Interestingly, two of the four patients included in this study without p16 positivity (patients 750, 865, 872, and 1002) had cancer recurrence and 1 of 4 had cancer-associated mortality. Three of four of these patients had negative anti-E7 values as predicted by the lack of high-risk HPV infection.

E7 oncoprotein positivity at the first visit was not useful to predict recurrence and survival (*p* = 1). Patients who had positive trending high-risk anti-E7 throughout the study were more likely to have worse clinical outcome with relapse of cancer or progression to mortality (*p* = .004). Of the 4 patients with an increasing anti-E7 trend, all had cancers progressing to mortality and 2 had cancer that recurred. A positive trending anti-E7 panel as a predictive test of recurrence or progression to mortality among p16+ HNC patients confers a 36.36% (95% CI 10.93% to 69.21%) sensitivity, 100% specificity (95% CI 88.06% to 100%), positive predictive value of 100%, and negative predictive value of 83.3% (72.60% to 86.62%).

## 4. Discussion

The presence of serum antibodies to E7 at the first clinical visit of HNSCC patients were measured for the first time recently and suggested that E7 may be a marker of cancer recurrence [19]. Additionally, it has been reported that E6 and E7 positivity correlates with better survival but worse tumor grade and stage [14]. However, no study to our knowledge has yet identified oncoprotein titer variation throughout the disease course to be useful for predicting HPV HNC clinical outcome [19–22].

**Figure 1:**
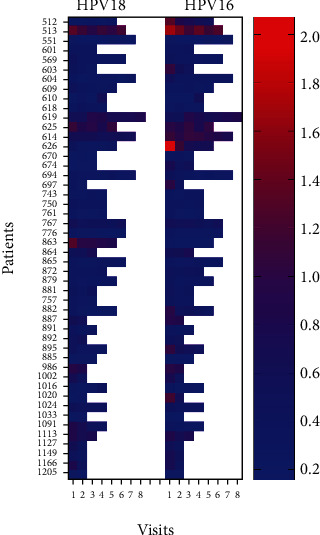
Heat map of anti-E7 ELISA values at consecutive clinical visits for HPV-18 and HPV-16 E7 antibodies. All patients with increasing trends on ELISA (patients 610, 619, 625, and 864) correlated with patient mortality. The p16 negative controls (patients 705, 865, and 872) are all seronegative for anti-E7 as predicted. Note that HPV-18 and HPV-16 were used to generate the E7 antigens but are not specific for these subtypes of HPV but rather indicative or reactivity to high-risk HPV E7 protein.

**Figure 2:**
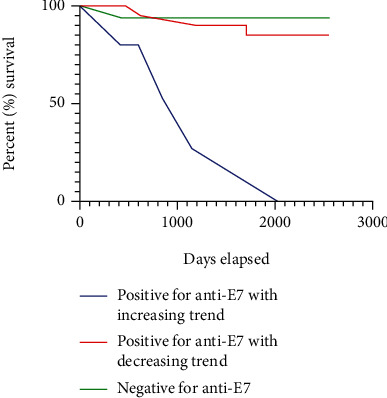
Survival data of patients differentiated by anti-E7 trend. The percent survival in each group following treatment is shown. Positive for anti-E7 with increasing trend *n* = 4, positive for anti-E7 with decreasing trend *n* = 19, negative for anti-E7 *n* = 25. Two of four patients suffered recurrence, and all four patients suffered mortality in the positive for anti-E7 with increasing trend group by day 2035.

**Figure 3:**
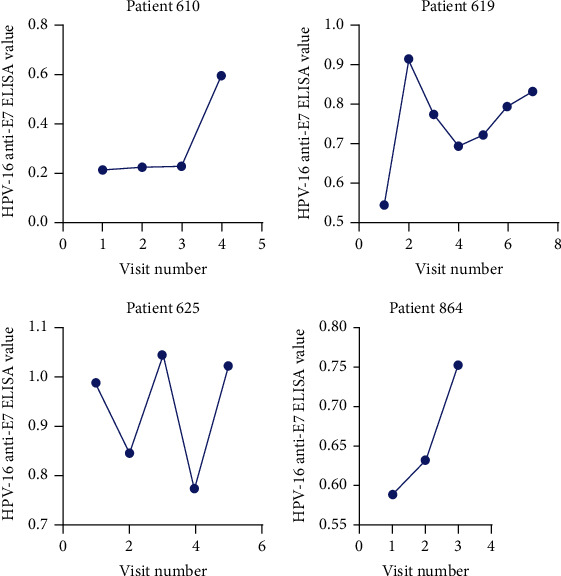
HPV-16 anti-E7 ELISA trends in patients with increasing ELISA values. All patients suffered mortality following treatment, and patients 610 and 625 suffered relapse following treatment. Note that HPV-16 was used to generate the E7 antigen but is not specific for this subtype of HPV but rather indicative or reactivity to high-risk HPV E7 protein.

Our data did not demonstrate the same trend of E7 oncoprotein positivity at the first visit to be useful to predict recurrence and survival (*p* = 1). This is likely because our study population was known HPV-associated HNC who should have elevated E7 in the bloodstream before undergoing extensive treatment rather than the study population of all HNC patients used in previous studies where HPV-associated HNC confers better survival. For the HPV-associated HNC population, our data demonstrates that an increasing anti-E7 trend throughout the course of treatment predicts mortality (*p* = .0007) and recurrence or progression to mortality (*p* = .004) with 100% specificity. The increasing anti-E7 trend for patients experiencing relapse and mortality is illustrated by the contingency tables (Tables 1 and 2), the heat map (Figure 1), and relapsed patients 610 and 625 (Figures 2 and 3).

Our ELISA is not specific enough to distinguish between subtypes of high-risk HPV accurately because of cross-reactivity between other high-risk HPV E7 proteins present in the sera due to sequence similarity. HPV-18 and HPV-16 were used to produce E7 antigens because these are common subtypes of high-risk HPV; however, we have denoted throughout the paper that the reactivity is against high-risk HPV E7 instead of specific subtypes of HPV. Furthermore, patients positive for both anti-E7 HPV 18 and anti-E7 HPV 16 do not necessarily indicate true positivity for both antibodies because the antigens have over 40% sequence similarity.

Although this data is not representative of specific subtypes of HPV E7 present in the sera, as a predictive test for clinical relapse or progression to mortality trending high-risk anti-E7 among HPV+ HNC patients may have utility as a specificity of 100% makes the test ideal for ruling in the possibility of relapse and cancer mortality. This test may allow increased disease activity to be identified, and patients may benefit from more aggressive treatment. The sensitivity of 36.36% is relatively low and makes this a poor screening test, especially for patients without high-risk HPV-associated cancer.

This novel trend will be useful to future studies that expand the sample size to introduce more biologic variability. Because of the ability of anti-E7 to predict clinical outcome and thus guide treatment, it should be considered as a target for high-risk HPV-associated HNC patient monitoring. Previous data is limited regarding direct ELISA or E7 in sera, although the clinical impact of a noninvasive test for stratifying outcomes is large. The use of novel biomarkers like E7 in conjunction with classical cancer staging techniques is critical to deliver high-quality tailored care. Further research comparing the prognostic efficacy of circulating E7 nucleic acid at sequential clinical visits with E7 antibodies would be impactful. This data is important to further our understanding of HPV serology, non-invasively monitor persistent or occult tumors, and will help to create personalized cancer treatments in the future.

## 5. Conclusion

High-risk human papillomavirus-associated head and neck cancer is an increasing issue with significant clinical outcome heterogeneity. Trending anti-E7 via liquid biopsy is strongly predictive of cancer recurrence or progression with mortality (*p* = .004). Measuring E7 oncoprotein at consecutive clinical visits may be a highly specific way to rule in cancer recurrence or fatal disease course, thus guiding head and neck cancer therapy.

## Figures and Tables

**Table 1:** E7 antibody trend predicts mortality in head and neck cancer.

		Number of patients (%)	Mortality	No mortality	*P-*value
Overall		48			
Lost to follow-up		5			
Patients available for mortality analysis		43	9	34	

Age	**<60**	24 (56)	6	18	
	**≥60**	19 (44)	3	16	.677 (NS)

Sex	**Male**	37 (86)	7	30	
	**Female**	6 (14)	2	4	.589 (NS)

Race	**White**	41 (95)	8	33	
	**African American**	2 (5)	1	1	.378 (NS)

Stage at diagnosis	**1**	1 (2)	1	0	
	**2**	3 (7)	0	3	
	**3**	6 (14)	4	2	
	**4**	33 (77)	4	29	.006(∗∗)

P16 +	**Yes**	39 (91)	8	31	
	**No (control)**	4 (9)	1	3	1 (NS)

E7 antibody trend among P16 positive patients	**Positive with increasing trend**	4 (10)	4	0	
	**Positive with decreasing trend**	19 (50)	3	16	
	**Negative**	16 (40)	1	15	.0007(∗∗∗)

Contingency tables of patient demographics at the time of diagnosis, stage, HPV relevant markers, and E7 trend are shown to compare HNC patients with mortality and patients with no mortality. The Fisher's exact test is used for the 2 × 2 contingency tables, and the Fisher's exact test with Freeman-Halton extension is added to the larger contingency table to show the significance level of observed differences (NS = not significant, ∗∗ = *p* ≤ .01, ∗∗∗ = *p* ≤ .001).

**Table 2:** E7 antibody trend predicts cancer recurrence or progression to mortality among HPV-associated HNC patients.

		Number of patients (%)	Cancer recurrence or progression to mortality	No cancer recurrence or progression to mortality	*P*-value
p16+ HNC patients		39	11	27	

E7 antibody trend among p16 positive patients	**Positive with increasing trend**	4 (10)	4	0	
	**Positive with decreasing trend**	19 (50)	5	14	
	**Negative**	16 (40)	2	14	*P* = .004(∗∗)

Contingency table of HPV-associated HNC patients' immune response trend in consecutive clinical visits throughout the treatment. The Fisher's exact test with Freeman-Halton extension is used for significance testing (∗∗ = *p* ≤ .01).

## Data Availability

The clinical data of HNC patients used to support the findings of this study are included within the supplementary information file.

## Supplementary Materials

Supplementary Materials Supplementary Table 1. The demographic and clinical characteristics of head and neck cancer patients. The patient identification number, age at diagnosis, cancer description, p16 status, stage at diagnosis, sex, race, survival status, and overall survival days are shown. All patients had cancers of the oropharynx apart from patients 881 and 891 which were denoted as having non-oropharynx cancers specifically. If the patient was alive at the time of data collection the survival days are shown up to that interval. Supplementary Table 2. Treatment. Patient study identification number, therapy type, therapy details, and day from day of diagnosis are shown. Supplementary Table 3. Patient mortality and recurrence by E7 trend following treatment. Patients entirely negative for anti-E7 HPV-16 and HPV-18, Patients with at least one increasing anti-E7 trend for HPV-16 or HPV-18, and Patients with at least one decreasing anti-E7 trend for HPV-16 or HPV-18 is shown following treatment. Highlighted in red are patients suffering from cancer recurrence, purple font is indicative of patients that suffered from mortality, and patients denoted with (∗) is indicative of p16 negative status. Note that HPV-18 and HPV-16 was used to generate the E7 antigens but are not specific for these subtypes of HPV but rather indicative or reactivity to high-risk HPV E7 protein.
