# Supplementary material for: Trending Anti-E7 Serology Predicts Mortality and Recurrence of HPV-Associated Cancers of the Oropharynx
Source: J Oncol. 2022 Sep 26;2022:3107990. doi: 10.1155/2022/3107990 (PMC9529406; doi:10.1155/2022/3107990)
Supplement: Supplementary Materials — Supplementary Table 1. The demographic and clinical characteristics of head and neck cancer patients. The patient identification number, age at diagnosis, cancer description, p16 status, stage at diagnosis, sex, race, survival status, and overall survival days are shown. All patients had cancers of the oropharynx apart from patients 881 and 891 which were denoted as having non-oropharynx cancers specifically. If the patient was alive at the time of data collection the survival days are shown up to that interval. Supplementary Table 2. Treatment. Patient study identification number, therapy type, therapy details, and day from day of diagnosis are shown. Supplementary Table 3. Patient mortality and recurrence by E7 trend following treatment. Patients entirely negative for anti-E7 HPV-16 and HPV-18, Patients with at least one increasing anti-E7 trend for HPV-16 or HPV-18, and Patients with at least one decreasing anti-E7 trend for HPV-16 or HPV-18 is shown following treatment. Highlighted in red are patients suffering from cancer recurrence, purple font is indicative of patients that suffered from mortality, and patients denoted with (∗) is indicative of p16 negative status. Note that HPV-18 and HPV-16 was used to generate the E7 antigens but are not specific for these subtypes of HPV but rather indicative or reactivity to high-risk HPV E7 protein. [file 3107990.f1.zip › Supplementary Table1.pdf]

Supplementary table 1: The demographic and clinical characteristics of head and neck cancer patients

Patient identification number	Age at primary diagnosis	Cancer Description	p16 Status	Stage	Sex	Primary Race	Status	Overall survival days
512	60	MALIGNANT NEOPLASM OF BASE OF TONGUE	p16 positive	4	Male	White	Alive	2546
513	49	MALIGNANT NEOPLASM OF LINGUAL TONSIL	p16 positive	4	Male	White	Alive	2551
551	53	MALIGNANT NEOPLASM OF TONSIL	p16 positive	4	Male	White	Alive	2560
569	61	MALIGNANT NEOPLASM OF TONSIL	p16 positive	3	Female	White	Alive	2454
601	50	MALIGNANT NEOPLASM OF OROPHARYNX, UNSPECIFIED	p16 positive	4	Male	White	Alive	2461
603	62	MALIGNANT NEOPLASM OF TONSIL	p16 positive	3	Male	White	Dead	620
604	67	MALIGNANT NEOPLASM OF BASE OF TONGUE	p16 positive	4	Male	White	Alive	2433

609	67	MALIGNANT NEOPLASM OF TONSIL	p16 positive	4	Male	White	Alive	2417
610	59	MALIGNANT NEOPLASM OF BASE OF TONGUE	p16 positive	4	Male	White	Dead	428
614	61	MALIGNANT NEOPLASM OF TONSIL	p16 positive	4	Male	White	Alive	2392
618	66	MALIGNANT NEOPLASM OF TONSIL	p16 positive	3	Male	White	Lost to follow- up	644
619	59	MALIGNANT NEOPLASM OF TONSIL	p16 positive	3	Female	White	Dead	1150
625	56	MALIGNANT NEOPLASM OF TONSIL	p16 positive	3	Female	African American	Dead	832
626	61	MALIGNANT NEOPLASM OF BASE OF TONGUE	p16 positive	4	Male	White	Alive	2340
670	59	MALIGNANT NEOPLASM OF BASE OF TONGUE	p16 positive	2	Male	White	Alive	2238
674	61	MALIGNANT NEOPLASM OF TONSIL	p16 positive	4	Male	White	Alive	2345

694	52	MALIGNANT NEOPLASM OF BASE OF TONGUE	p16 positive	4	Male	White	Alive	2306
697	45	MALIGNANT NEOPLASM OF BASE OF TONGUE	p16 positive	4	Male	White	Lost to follow- up	459
743	55	SCC METASTATIC TO RIGHT NECK, UNKOWN PRIMARY	p16 positive	4	Male	White	Alive	2230
750	49	MALIGNANT NEOPLASM OF TONSIL	p16 negative	4	Male	White	Dead	583
757	57	MALIGNANT NEOPLASM OF TONSIL	p16 positive	4	Female	White	Dead	439
761	58	MALIGNANT NEOPLASM OF TONSIL	p16 positive	4	Male	White	Alive	2155
767	64	MALIGNANT NEOPLASM OF TONSIL AND MALIGNANT NEOPLASM OF LOWER LOBE, BRONCHUS OR LUNG	p16 positive	3	Male	White	Dead	1708

776	61	MALIGNANT NEOPLASM OF TONSIL	p16 positive	4	Male	White	Alive	2096
863	55	MALIGNANT NEOPLASM OF TONSIL	p16 positive	4	Male	White	Alive	2030
864	54	MALIGNANT NEOPLASM OF BASE OF TONGUE	p16 positive	4	Male	White	Dead	2035
865	65	MALIGNANT NEOPLASM OF TONSIL	p16 negative	4	Male	White	Alive	2052
872	58	MALIGNANT NEOPLASM OF TONSIL	p16 negative	4	Male	White	Alive	2000
879	63	MALIGNANT NEOPLASM OF TONSIL	p16 positive	2	Male	White	Alive	1953
881	59	SCC OF LARYNX WITH TRUE VOCAL CORD INVOLVMENT (NON-OROPHARYNX)	p16 positive	3	Male	African American	Alive	1961

882	56	MALIGNANT NEOPLASM OF BASE OF TONGUE	p16 positive	4	Male	White	Alive	1942
887	51	MALIGNANT NEOPLASM OF TONSIL	p16 positive	4	Male	White	Alive	1905
891	57	SCC ORAL TONGUE (NON-OROPHARYNX)	p16 positive	4	Female	White	Alive	1942
892	55	MALIGNANT NEOPLASM OF OTHER SPECIFIED SITES OF OROPHARYNX	p16 positive	3	Female	White	Lost to follow- up	599
895	59	SCC UNKOWN PRIMARY, METASTATIC TO LEFT NECK	p16 positive	4	Male	White	Alive	1901
985	58	MALIGNANT NEOPLASM OF TONSIL	p16 positive	4	Male	White	Alive	1877
986	49	SCC UNKOWN PRIMARY, METASTATIC TO LEFT NECK	p16 positive	4	Male	White	Lost to follow- up	165

1002	61	MALIGNANT NEOPLASM OF TONSIL	p16 negative	4	Female	White	Lost to follow-up	234
1016	56	MALIGNANT NEOPLASM OF TONSIL	p16 positive	4	Male	White	Alive	1788
1020	61	MALIGNANT NEOPLASM OF BASE OF TONGUE	p16 positive	4	Male	White	Alive	1793
1024	66	MALIGNANT NEOPLASM OF TONSIL	p16 positive	4	Male	White	Alive	1740
1033	60	MALIGNANT NEOPLASM OF LATERAL WALL OF OROPHARYNX	p16 positive		Male	White	Alive	1724
1091	65	MALIGNANT NEOPLASM OF BASE OF TONGUE AND MALIGNANT NEOPLASM OF KIDNEY	p16 positive	4	Male	White	Alive	1702

1113	54	MALIGNANT NEOPLASM OF TONSIL AND MALIGNANT NEOPLASM OF LOWER LOBE, BRONCHUS OR LUNG	p16 positive	4	Female	African American	Lost to follow- up	357
1127	71	MALIGNANT NEOPLASM OF BASE OF TONGUE	p16 positive	4	Male	White	Alive	1637
1149	48	MALIGNANT NEOPLASM OF BASE OF TONGUE	p16 positive	4	Male	White	Alive	1576
1166	76	MALIGNANT NEOPLASM OF TONSIL	p16 positive	1	Male	White	Dead	1190
1205	65	METASTIC SCC DIAGNOSED ON LYMPH NODE BIOPSY WITH LEFT BASE OF TONGUE NODULE ON POSITRON EMISSION TOMOGRAPHY	p16 positive	2	Male	White	Alive	1467

Supplementary table 1: The patient identification number, age at diagnosis, cancer description, p16 status, stage at diagnosis, sex, race, survival status, and overall survival days are shown. All patients had cancers of the oropharynx apart from patients 881 and 891

which were denoted as having non-oropharynx cancers specifically. If the patient was alive at the time of data collection the survival days are shown up to that interval.
