# Supplementary material for: Trending Anti-E7 Serology Predicts Mortality and Recurrence of HPV-Associated Cancers of the Oropharynx
Source: J Oncol. 2022 Sep 26;2022:3107990. doi: 10.1155/2022/3107990 (PMC9529406; doi:10.1155/2022/3107990)
Supplement: Supplementary Materials — Supplementary Table 1. The demographic and clinical characteristics of head and neck cancer patients. The patient identification number, age at diagnosis, cancer description, p16 status, stage at diagnosis, sex, race, survival status, and overall survival days are shown. All patients had cancers of the oropharynx apart from patients 881 and 891 which were denoted as having non-oropharynx cancers specifically. If the patient was alive at the time of data collection the survival days are shown up to that interval. Supplementary Table 2. Treatment. Patient study identification number, therapy type, therapy details, and day from day of diagnosis are shown. Supplementary Table 3. Patient mortality and recurrence by E7 trend following treatment. Patients entirely negative for anti-E7 HPV-16 and HPV-18, Patients with at least one increasing anti-E7 trend for HPV-16 or HPV-18, and Patients with at least one decreasing anti-E7 trend for HPV-16 or HPV-18 is shown following treatment. Highlighted in red are patients suffering from cancer recurrence, purple font is indicative of patients that suffered from mortality, and patients denoted with (∗) is indicative of p16 negative status. Note that HPV-18 and HPV-16 was used to generate the E7 antigens but are not specific for these subtypes of HPV but rather indicative or reactivity to high-risk HPV E7 protein. [file 3107990.f1.zip › Supplementary Table2.pdf]

Supplementary Table 2: Treatment

Patient Study ID	Therapy type	Therapy Details	Therapy day from day of diagnosis
512	Chemo / Imuno / etc	Cisplatin	69
512	Radiation	curative radiation to the Bottom of Tongue	82
513	Chemo / Imuno / etc	Cisplatin	76
513	Radiation	curative radiation to the Bottom of Tongue	83
551	Surgery	cervical node FNA	20
551	Surgery	left tonsil biopsy	38
551	Chemo / Imuno / etc	Cisplatin	104
551	Radiation	radiation to tonsil and neck	111
553	Surgery	FNA of right neck lymph node	0
553	Chemo / Imuno / etc	Cetuximab	68
553	Radiation	radiation to tonsil and lymph nodes	76
569	Surgery	left tonsil biopsy	0
569	Radiation	concurrent radiation to left neck	109
569	Chemo / Imuno / etc	Cetuximab	110
601	Surgery	biopsy of right neck mass	0
601	Chemo / Imuno / etc	cisplatin	103
601	Radiation	concurrent radiation to neck and toinsil	105
603	Surgery	right tonsillar biopsy	0
603	Chemo / Imuno / etc	Cisplatin	63

603	Radiation	concurrent radiation to neck and tonsil	84
603	Chemo / Imuno / etc	paclitaxel	253
603	Chemo / Imuno / etc	cetuximab	336
603	Chemo / Imuno / etc	Nivolumab	430
603	Chemo / Imuno / etc	Docetaxel	539
603	Chemo / Imuno / etc	5 FU	568
603	Radiation	palliative radiation to mediastinum	583
603	Radiation	palliative radiation to brain	595
604	Surgery	right neck mass FNA	0
604	Chemo / Imuno / etc	cisplatin	82
604	Radiation	radiation to neck mass	89
604	Surgery	L1 vertebral body, core biopsy	1419
604	Chemo / Imuno / etc	nivolumab	1837
604	Chemo / Imuno / etc	paclitaxel/cetuximab	1949
604	Chemo / Imuno / etc	carboplatin/5FU	2306
609	Surgery	Left tonsil biopsy	0
609	Radiation	radiation to left tonsil	67
609	Surgery	Left lower wedge resection with lymph node dissection	708
610	Radiation	concurrent radiation	61
610	Chemo / Imuno / etc	cisplatin	61

610	Chemo / Imuno / etc	Carboplatin	94
610	Chemo / Imuno / etc	paclitaxel	385
610	Chemo / Imuno / etc	Pembrolizumab	419
613	Surgery	Right tonsil mass biopsy	0
613	Chemo / Imuno / etc	Cisplatin	86
613	Radiation	radiation to the right tonsil	98
614	Surgery	FNA right neck mass	0
614	Chemo / Imuno / etc	Cisplatin	19
614	Chemo / Imuno / etc	Cetuximab	26
614	Chemo / Imuno / etc	Carboplatin and Paclitaxel	62
614	Radiation	chemoradiation to right tonsil	68
618	Surgery	Left tonsil biopsy	0
618	Chemo / Imuno / etc	Cisplatin	64
618	Radiation	radiation to left tonsil	70
619	Surgery	right tonsil biopsy	0
619	Chemo / Imuno / etc	Cisplatin	106
619	Radiation	radiation to tonsil	112
625	Surgery	left tonsil biopsy	0
625	Chemo / Imuno / etc	Cisplatin	90
625	Radiation	radiation to tonsil and Base of Tongue	97

625	Surgery	liver lesion biopsy	789
626	Surgery	right neck mass FNA	14
626	Chemo / Imuno / etc	high dose cisplatin	50
626	Radiation	radiatin to base of tongue 70 GY	71
670	Surgery	Right modified radical neck dissection levels II through IV	0
670	Chemo / Imuno / etc	Cetuximab	64
670	Radiation	concurrent radiation	70
673	Surgery	left tonsillectomy	0
674	Surgery	right palate biopsy	0
674	Surgery	right side neck mass FNA	0
674	Chemo / Imuno / etc	Cisplatin	130
674	Radiation	radiation to right tonsil	130
674	Surgery	liver biopsy	232
674	Chemo / Imuno / etc	cetuximab, carboplatin, 5-FU	316
674	Chemo / Imuno / etc	Pembrolizumab	340
675	Surgery	left tonsil biopsy	0
675	Chemo / Imuno / etc	cisplatin	99
675	Radiation	radiation to left tonsil	108
694	Surgery	FNA of Cervical Lymph Node	0

694	Surgery	Direct Laryngoscopy with Biopsy	51
694	Chemo / Imuno / etc	Cisplatin	125
694	Radiation	radiation to base of tongue	127
697	Surgery	right base of tongue biopsy	0
697	Surgery	ultrasound guided biopsy of left neck lymph node	16
697	Chemo / Imuno / etc	Cisplatin	69
697	Radiation	external beam radiation therapy	72
743	Surgery	biopsy of right neck lymph node	0
743	Surgery	nasopharynx, piriform sinus, base of tounge, and tonsil biopies	12
743	Chemo / Imuno / etc	Cisplatin	63
743	Radiation	Radiation to the neck	63
750	Surgery	left tonsil biopsy	28
750	Chemo / Imuno / etc	high-dose Cisplatin plus Lapatinib/placebo	132
750	Radiation	radiation to left neck mass	158
750	Surgery	ultrasound-guided core biopsy of right hepatic lobe mass	270

750	Chemo / Imuno / etc	Nivolumab +/- Ipilimumab clinical trial	382
750	Chemo / Imuno / etc	Carboplatin and Paclitaxel	466
750	Chemo / Imuno / etc	cetuximab plus or minus pablociclib	520
757	Surgery	Left neck mass biopsy	0
757	Chemo / Imuno / etc	cetuximab	89
757	Radiation	radiation to neck mass	96
761	Surgery	FNA Left Neck Mass	0
761	Surgery	Tonsillectomy - Right and left	8
761	Chemo / Imuno / etc	Cisplatin	100
767	Surgery	right tonsil biopsy	0
767	Surgery	right hilar lymph node, FNA biopsy	0
767	Chemo / Imuno / etc	high dose cisplatin	54
767	Radiation	radiation to tonsil and neck	72
767	Surgery	Tongue, Right, Biopsy	273
776	Surgery	right lateral tonsil biopsy	0
776	Chemo / Imuno / etc	Cisplatin	82
776	Radiation	radiation to tonsil and adenopathy	95
776	Surgery	R paratracheal LN, FNA	879
776	Chemo / Imuno / etc	Pembrolizumab	1986

863	Chemo / Imuno / etc	Cisplatin	69
863	Radiation	Radiation	75
864	Surgery	direct laryngoscopy	0
864	Chemo / Imuno / etc	high dose cisplatin	69
864	Radiation	radiation to base of tongue and lymph nodes	94
864	Surgery	lymph node, right hilar, FNA	1196
864	Chemo / Imuno / etc	nivolumab	1569
864	Chemo / Imuno / etc	Pembrolizumab	1989
865	Surgery	FNA of left neck lymph node	0
865	Chemo / Imuno / etc	Cisplatin	107
865	Radiation	radiation to tonsil and lymph nodes	113
865	Surgery	bronchoscopy and biopsy left upper lobe	302
865	Radiation	radiation to left upper lobe	329
872	Surgery	tonsillectomy	0
872	Chemo / Imuno / etc	High dose Cisplatin	74
872	Radiation	concurrent radiation	81
879	Chemo / Imuno / etc	Cisplatin	83
879	Radiation	Radiation tonsil and neck	89
882	Surgery	tongue biopsy	0

882	Surgery	base of tongue biopsy	17
882	Radiation	75 Gy in 35 fractions using IMRT	21
882	Chemo / Imuno / etc	Cisplatin-concurrent radiation	72
884	Surgery	left BOT biopsy	0
884	Chemo / Imuno / etc	cisplatin	57
884	Radiation	radiation	76
885	Surgery	Left oropharyngeal biopsy	0
885	Chemo / Imuno / etc	cisplatin	98
885	Radiation	radiation	103
887	Surgery	right tonsil biopsy	0
887	Chemo / Imuno / etc	cisplatin	48
887	Radiation	radiation	63
891	Surgery	ventral tongue biopsy	0
891	Surgery	right hemiglossectomy with right and left neck dissection	36
891	Chemo / Imuno / etc	Cisplatin	114
891	Radiation	radiation	116
892	Surgery	left arytenoid biopsy	0
892	Chemo / Imuno / etc	High dose Cisplatin +- Lapatanib (RTOG)	127
892	Radiation	concurrent radiation	127
895	Surgery	Left submanibular lymph node biopsy	0

895	Chemo / Imuno / etc	Cisplatin	63
895	Radiation	radiation	72
900	Surgery	right tonsil biopsy	0
985	Surgery	right tonsil, biopsy	0
985	Chemo / Imuno / etc	cisplatin	88
985	Surgery	lymph nodes, left neck, Levels II-IV, neck dissection	319
986	Surgery	FNA right neck mass	0
986	Surgery	excision of right and left tonsil	12
986	Surgery	neck lymph node dissection	50
986	Chemo / Imuno / etc	Cisplatin	110
986	Radiation	radiation to right neck	118
987	Surgery	Right neck mass FNA	0
987	Surgery	right neck mass biopsy	4
987	Surgery	tonsil biopsy	11
987	Chemo / Imuno / etc	high dose cisplatin	32
987	Chemo / Imuno / etc	avelumab	85
987	Radiation	radiation	97
987	Chemo / Imuno / etc	maintenance avelumab/placebo	463
1001	Surgery	left base of tongue biopsy	0
1001	Surgery	left hilar lymph node FNA	0

1001	Chemo / Imuno / etc	Pembrolizumab	233
1001	Surgery	Lingula, Bronchoalveolar Lavage	351
1001	Chemo / Imuno / etc	paclitaxel and carboplatin	400
1002	Surgery	right tonsil excision	0
1002	Chemo / Imuno / etc	high-dose cisplatin	84
1002	Radiation	curative radiation to tonsil and lymph nodes	100
1016	Surgery	right tonsil biopsy	0
1016	Radiation	concurrent radiation	78
1016	Chemo / Imuno / etc	High dose Cisplatin	78
1020	Surgery	left base of tongue biopsy	0
1020	Chemo / Imuno / etc	high-dose cisplatin	68
1020	Radiation	radiation to tonsil and adenopathy	86
1024	Surgery	left tonsil excision	0
1024	Chemo / Imuno / etc	high-dose cisplatin	71
1024	Radiation	radiation to tonsil and adenopathy	71
1033	Surgery	right lateral oropharyngeal wall biopsy	0
1033	Radiation	concurrent radiation	69
1033	Chemo / Imuno / etc	high-dose cisplatin	69

1033	Surgery	right neck dissection	306
1091	Surgery	left base of tongue biopsy	0
1091	Chemo / Imuno / etc	cetuximab	80
1091	Radiation	radiation	80
1112	Surgery	left mass biopsy	0
1112	Chemo / Imuno / etc	cisplatin +/- avelumab	68
1112	Radiation	radiation	75
1113	Surgery	right lower lobe mass FNA	0
1113	Surgery	right tonsil biopsy	0
1113	Chemo / Imuno / etc	cisplatin	73
1113	Radiation	radiation	81
1113	Surgery	Liver mass biopsy	329
1127	Surgery	biopsy right base tongue	0
1127	Chemo / Imuno / etc	Avelumab/placeboplus high dose Cisplatin	78
1127	Radiation	radiation	93
1127	Chemo / Imuno / etc	maintenance avelumab/placebo	436
1149	Surgery	Left base of tongue biopsy	0
1149	Chemo / Imuno / etc	High dose Cisplatin	77
1149	Radiation	concurrent radiation	77
1150	Surgery	left tonsil biopsy	0
1150	Chemo / Imuno / etc	cisplatin	66
1150	Radiation	radiation	90

1166	Surgery	FNA of right neck mass	0
1166	Chemo / Imuno / etc	Cisplatin	114
1166	Radiation	radiation	114
1166	Surgery	FNA of right submandibular mass	211
1166	Surgery	BOT biopsy	1093
1205	Surgery	right cervical lymph node biopsy	0
1205	Chemo / Imuno / etc	high-dose cisplatin	75
1205	Chemo / Imuno / etc	concurrent radiation	75

Supplementary Table 2: Patient study identification number, therapy type, therapy details, and day from day of diagnosis are shown.
