# Supplementary material for: Trending Anti-E7 Serology Predicts Mortality and Recurrence of HPV-Associated Cancers of the Oropharynx
Source: J Oncol. 2022 Sep 26;2022:3107990. doi: 10.1155/2022/3107990 (PMC9529406; doi:10.1155/2022/3107990)
Supplement: Supplementary Materials — Supplementary Table 1. The demographic and clinical characteristics of head and neck cancer patients. The patient identification number, age at diagnosis, cancer description, p16 status, stage at diagnosis, sex, race, survival status, and overall survival days are shown. All patients had cancers of the oropharynx apart from patients 881 and 891 which were denoted as having non-oropharynx cancers specifically. If the patient was alive at the time of data collection the survival days are shown up to that interval. Supplementary Table 2. Treatment. Patient study identification number, therapy type, therapy details, and day from day of diagnosis are shown. Supplementary Table 3. Patient mortality and recurrence by E7 trend following treatment. Patients entirely negative for anti-E7 HPV-16 and HPV-18, Patients with at least one increasing anti-E7 trend for HPV-16 or HPV-18, and Patients with at least one decreasing anti-E7 trend for HPV-16 or HPV-18 is shown following treatment. Highlighted in red are patients suffering from cancer recurrence, purple font is indicative of patients that suffered from mortality, and patients denoted with (∗) is indicative of p16 negative status. Note that HPV-18 and HPV-16 was used to generate the E7 antigens but are not specific for these subtypes of HPV but rather indicative or reactivity to high-risk HPV E7 protein. [file 3107990.f1.zip › Supplementary Table3.pdf]

Supplementary Table 3: patient mortality and recurrence by E7 trend following treatment		
Patients entirely negative for anti-E7 HPV-16 and HPV-18	Patients with at least one increasing anti-E7 trend for HPV-16 or HPV-18	Patients with at least one decreasing anti-E7 trend for HPV-16 or HPV-18
551	512	610
601	513	619
618	603	625
670	625	864
743	614	
761	626	
776	674	
872*	697	
879	767	
881	882	
891	887	
985	895	
1016	986	
1033	1002*	
1205	1091	
750*	1113	
865*	1127	
892	1149	
757	1166	
694		
863		
1024		
569		
604		
609		

Supplementary Table 3: patient mortality and recurrence by E7 trend: Patients entirely negative for anti-E7 HPV-16 and HPV-18, Patients with at least one increasing anti-E7 trend for HPV-16 or HPV-18, and Patients with at least one decreasing anti-E7 trend for

HPV-16 or HPV-18 is shown following treatment. Highlighted in red are patients suffering from cancer recurrence, purple font is indicative of patients that suffered from mortality, and patients denoted with (*) is indicative of p16 negative status. Note that HPV-18 and HPV-16 was used to generate the E7 antigens but are not specific for these subtypes of HPV but rather indicative or reactivity to high-risk HPV E7 protein.
